# Evaluating online family life education modules across family stages: a pilot study

**DOI:** 10.3389/fpubh.2026.1909217

**Published:** 2026-09-17

**Authors:** Emma L. Alexander, Krista Stewart, Hayley Poffenberger Taylor, AliceAnn Crandall

**Affiliations:** Department of Public Health, Brigham Young University, Provo, UT, United States

**Keywords:** digital education, family life education, family life stages, family well-being, older adults, young adults

## Abstract

**Objective:**

To evaluate whether online family life education (FLE) modules improved knowledge, attitudes, and confidence about key family health topics among young, middle, and older adults.

**Background:**

Family life education supports healthy familial relationships across generations. Traditionally, FLE has been offered in-person over multiple weeks. Online FLE could make it easier to reach families.

**Methods:**

The final sample was comprised of 164 adults (45% young adults, 45% middle adults, and 10% older adults). Ten online video modules (each ~10 min) were created by college students about a variety of family health topics. For each module, participants were asked to complete a pre-survey, watch the module, and then complete a post-survey. Data were analyzed in Stata 18.

**Results:**

Participants reported that the modules were clear, engaging, and well-organized. Participants showed significant improvements in knowledge and attitudes between the pre- and post-surveys for all modules, including 69 of the 75 the outcomes measured. Effect sizes were generally medium to large, with significant effect sizes ranging from 0.27 to 0.87. Long-term changes in family health were not significant.

**Conclusion:**

Short, online FLE modules can yield immediate gains in knowledge and attitudes across family health topics. Further refinement in methodology is needed to promote longer-term gains in family health.

**Implications:**

Brief, online FLE can strengthen knowledge, attitudes, and self-efficacy about family life issues across family life stages.

## Background

All families regularly encounter challenges which can place strain on relationships and overall family well-being, especially without a clear roadmap for addressing them. Some of these challenges include recovering from trauma, reproductive issues, loss of a job, relationship challenges, and so forth. To help families navigate these challenges, family life education (FLE) was developed to support families in cultivating healthy relationships across the lifespan (1). FLE is a strength-based, educational approach that equips individuals and families with the knowledge and skills necessary to enhance their own well-being and functioning (2).

The primary goal of FLE is to strengthen families through preventive education that builds skills needed to navigate everyday challenges. It does this by teaching families core skills such as communication, decision-making, managing self-esteem, understanding human development, and building healthy relationships, while also addressing broader societal issues like economics, work-family balance, parenting, sexuality, and gender (1). Collectively, these competencies are intended to strengthen both individual and family functioning by preparing families to navigate challenges across the lifespan. These skills are important because they help families manage everyday challenges as well as maintain these healthy habits over time. For instance, strong communication skills enable families to navigate conflicts effectively, while knowledge of age-specific human development provides guidance to parents at each stage of a child’s growth (3). Families that have access to FLE may be better equipped to handle the different life-stages families experience. Similarly, skills such as decision-making and self-esteem support individuals in making thoughtful choices and developing confidence, which can positively influence overall family interactions. FLE aims to strengthen individuals and families, prevent common social problems, and support optimal functioning across diverse contexts (4). Taken together, these principles set FLE up as a comprehensive framework for promoting healthy family systems and well-being across varying stages of life and environments. As interest in expanding access to FLE has grown, researchers and educators have increasingly explored new methods for delivering these resources to diverse families.

Traditionally offered in-person such as in schools and community centers, FLE has the potential to expand beyond in-person classroom settings. When the internet first became common in households, early research noted that it was becoming a rapidly growing medium for parenting information (5). Today, 34% of parents (42% of mothers and 22% of fathers) say they visit online communities where parents talk about raising children at least monthly (6). As artificial intelligence (AI) models become more proficient, individuals may increasingly turn to online support to help them with parenting and other family issues (7). Together, these developments suggest that families are increasingly seeking digital resources to obtain parenting information, encouraging new opportunities for delivering evidence-based FLE in accessible online formats. As more educational programs have moved online, asynchronous formats, learning environments where participants engage with materials on their own schedule rather than in real time, have become more important for accessible FLE (8). This flexible format allows families to engage with content at convenient times, which is especially valuable for those balancing work, caregiving, and other responsibilities (9). Because of this, having an asynchronous form of FLE will allow more families the chance to have access to FLE. Although this format may reduce opportunities for real-time interaction, it still supports meaningful learning across diverse family roles (10).

Although online education has expanded rapidly, prior research has not fully evaluated online Family Life Education as well as examined how different generations engage with these module formats. Additionally, little is known about whether online modules can produce intermediate learning or long-term family health improvements—thus representing a clear gap this study addresses. For example, relatively little research has examined whether brief, asynchronous FLE modules improve knowledge, attitudes, and self-efficacy related to family well-being across different adult age groups. Therefore, the purpose of this study was to examine the short-term and intermediate effects of a series of brief, asynchronous online FLE modules among young adults, middle-aged adults, and older adult participants.

### Family life education theory and practices

Family life education is built on foundational theories that explain how family’s function, interact, and develop over time. Based on family systems theory developed by Bowen (11) and expanded on by Brown & Errington (12), FLE is rooted in the idea that families operate as interconnected, dynamic systems that influence overall family well-being. Building on this foundation, effective FLE connects what individuals learn across different stages of life with how their families and communities shape them (13). Consistent with this framework, a fundamental principle to FLE is how people and families continually influence one another within their broader social and cultural environments (14). Earlier conceptualizations of FLE emphasized this interconnectedness. Arcus (15) explained that when these objectives were met, families were better able to prevent and manage problems, in turn contributing to a stronger and more stable society. Reflecting these theoretical foundations, FLE addresses families in different societal contexts, internal dynamics, growth, development across the lifespan, and parenting guidance (16). Additionally, this instruction is effective when it is tailored to the specific community or individual attending the class. Myers-Walls et al. (17) explained that for this reason it is important that the learner be an active partner with the FLE instructor.

FLE is designed to support both individuals and families across the entire lifespan, with the developmental needs of learners varying significantly by life stage. For instance, research demonstrates that adolescents benefit from FLE through measurable gains in communication, decision-making, and sexual health knowledge (18). Midlife adults show improvements in parental confidence, communication, and discipline practices through parent education programs (18). Likewise, older adults benefit from FLE programs focused on caregiving and health transitions, which support coping and adjustment during later-life changes (18). Benson & Donehower (19) explain that each life group engages with FLE differently because they bring distinct goals and motivations to the learning process. Taken together, these findings suggest that while FLE provides benefits across the lifespan, the developmental needs and motivations of participants vary by age, which highlights the need to evaluate these outcomes across diverse populations. Given these documented differences across the life course, this study seeks to understand how learning outcomes vary by age group in online FLE modules.

### Family life education venues: online vs. traditional settings

Many families face barriers to attending in-person FLE, including work schedules, caregiving responsibilities, and geographic distance. As society and technology have evolved, FLE has the potential to expand beyond traditional in-person classrooms (1, 5) to online asynchronous formats that allow for greater accessibility (8). In these environments, participants engage with materials on their own schedule, allowing for greater flexibility and independence. Research shows that asynchronous environments reduce scheduling barriers and support sustained engagement, particularly for individuals who cannot attend live events (10). This shift toward flexible delivery reflects ongoing efforts to make FLE more accessible to diverse populations.

Another type of online learning includes synchronous learning where participants can join online at the same time as other participants (20). Synchronous online learning provides real-time interaction and immediate feedback, but it can be difficult for families to attend consistently due to scheduling and technological barriers. Because the present study evaluates only asynchronous delivery, we focus this literature review on flexible, self-paced formats that allow participants to engage with content on their own time (10, 20).

Prior research comparing asynchronous and synchronous online formats suggests that asynchronous instruction may yield slightly stronger learning outcomes, a finding with important implications for FLE (21). For example, in an experiment comparing the two formats in an undergraduate microeconomics course, students in the asynchronous group scored significantly higher on the weekly assessment than those in the synchronous group (21). Collectively, these studies suggest that asynchronous online learning can improve accessibility while maintaining meaningful educational engagement, although evidence specific to FLE remains limited. Additional research supports the potential of online formats to produce measurable learning outcomes. Lee et al. (22) demonstrated that online learning behaviors analyzed with deep learning models can effectively predict student performance, suggesting that well-designed online environments can support meaningful engagement. Although systematic evaluation within FLE is still developing, these studies indicate that online formats—particularly asynchronous ones—can support strong learning outcomes when aligned with learner needs.

### Aims and hypotheses

Although online FLE has begun to address barriers to accessing FLE, online FLE has not been well-evaluated to examine how well these programs support families across multiple generations or how they influence knowledge, attitudes, and self-efficacy. Current research on online FLE is limited, notably regarding how multigenerational families (young adults, parents, and grandparents) engage with online modules and how much exposure is needed to produce meaningful improvements in family health. Little is known about differences in participation across generations, the extent to which a video module can lead to short-term gains in knowledge, or how many modules are required for measurable long-term changes. To address these gaps, this study examined a pilot online FLE curriculum with the following three aims: (1) Evaluate generational patterns of participation in online FLE by assessing what proportion of each generation (young, middle, and adult adults) engaged with online FLE modules, how many they completed, and whether participation differed by age. We hypothesized that young adults would participate at the highest rates. (2) Investigate whether viewing a single module produces short-term gains in knowledge and skill development and identify which modules generated the strongest improvements across generations. We hypothesized that there would be measurable short-term increases in knowledge, attitudes, and self-efficacy relating to each module. (3) Examine whether participating in online FLE resulted in longer-term improvements in family health. We hypothesized that participating in at least one FLE module would improve family health, with a dose–response relationship (e.g., those who participated in more FLE modules would have better family health compared to baseline). This dose–response pattern would be expected because prior research has found that FLE skills strengthen through repeated exposure and practice (23).

Because this paper reports on the results of a pilot study, further testing with comparison or control groups will be necessary to establish the short, intermediate, and long-term effects of online FLE. This paper only reports associations found in the study. The primary contribution of the current study is to examine the feasibility of short, asynchronous FLE modules across life stages.

## Method

### Sample and procedures

Participants for this study included 164 young adults, middle adults, and older adults. They were recruited from a larger three-generation on family health comprised of grandparents, parents, and young adult children/grandchildren (24, 25). The 164 participants represented 116 family triads (71 families with one participant throughout the study, 42 families with two members of the family triad who participated in one or more surveys, and three (3) families with three members of the family triad who participated in one or more surveys). Participants were recruited into the prior study through the young adult, who lived in a college town in the United States Intermountain West. In total, 481 people participated in the original three-generation study. In August 2024, all participants from the original study were invited (via e-mail and text) to join a private Facebook group created specifically for this project; 260 people joined the Facebook group (54%). After joining the group, participants were asked to complete a baseline survey assessing their existing knowledge, skills, and needs related to the upcoming FLE modules (128 participants completed the baseline survey). Participation in the baseline survey was not required to participate in the FLE modules. FLE modules were posted on the Facebook page beginning in October 2024 and a new module was posted every 3–8 weeks, with the last module posted in October 2025. All previously released modules remained accessible throughout the 12-month period. Participants could choose which modules to participate in. Every module included both a pre-test and a post-test. The pre-test contained a link to the video module. The video module included a password which participants had to enter to take the post-test to ensure that they had watched the module before taking the post-test. In total, 164 people from the original study (34% response rate) completed the baseline survey and/or at least one FLE module. Participants who completed at least one survey were invited to complete an end-of-study survey approximately 1 month after the last module was posted (November 2025). Of those who participated in the study, there was no difference in number of modules completed based on sex, race, or education. As outlined in the Results section below, few older adults participated in the current study. Further analysis of differences by demographic characteristics is difficult since not all participants completed the baseline or end-of-study surveys.

Incentives were provided in the form of $5 Amazon e-gift cards for each completed survey (baseline, pre, and post); participants in the end-of-study survey were entered into a drawing to receive one of five $20 Amazon gift cards. All recruitment procedures, study materials, and incentives were reviewed and approved by the university’s Institutional Review Board prior to data collection.

### Development of FLE modules

Table 1 contains a list of the 10 FLE modules offered and the number of participants who participated in each module. Module topics were selected based on the family needs identified in the prior study as well as participant requests for module topics in the baseline survey. Each module was offered as an asynchronous video with video lengths ranging from 8–12 min. Each video included visual aids, discussion prompts, and key takeaways. The modules were designed to be “stand alone;” the modules did not build on each other, but each provided brief coverage on a specific topic relevant to family life. Thus, participants could pick and choose modules to engage in based on their current needs.

**Table 1:** Family life education modules.

Topic	Video Length (Minutes)	Description
Navigating reproductive health conversations with family	12:00	Teaches how to foster open and comfortable conversations about reproductive health while emphasizing the importance of starting early and maintaining these discussions across different stages of life.
Positive adult experiences	11:25	Covers how positive adult experiences, such as strong friendships or hobbies, can help balance the effects of adverse childhood experiences.
Healthy habits	8:36	Focuses on modeling healthy physical, dietary, and emotional habits to create an environment that allows children to thrive.
Technology habits in the family	7:30	Covers setting boundaries for technology use, encouraging open communication, providing screen time guidelines, and discussing age-specific technology use.
Family mental health	9:49	Teaches how to recognize signs of poor mental health and support family members through available mental health resources.
Healing from trauma	11:42	Covers how trauma, such as PTSD or ACEs, can be addressed through supportive family environments and provides strategies that may promote healing.
Parenting teens	7:01	Focuses on the challenges teens face, emphasizing mental health struggles and the need for independence while providing strategies for parents navigating this stage of life.
Maintaining family Relationships across the lifespan	8:24	Focuses on what makes families healthy across different stages of life, including parenting styles, developmental needs, and maintaining connection over long distances.
Handling sensitive issues	11:46	Explores how to navigate sensitive issues by practicing active listening, respecting cultural differences, and finding common ground.
Adult sibling rivalry	12:33	Covers how adult sibling rivalry impacts family dynamics and offers strategies to strengthen sibling relationships.

The videos were developed by a team of undergraduate and graduate students majoring in public health, family science, and other life sciences. As part of a semester-long project for a Families and Health undergraduate and graduate student course, students were required to develop a family life education module. The course was required for all first year Master of Public Health (MPH) students and was an elective for undergraduate public health majors and graduate students from other majors (73% of students in the course were graduate students; 16% of the graduate students came from other majors outside of public health). Students worked in teams of 3–5 students to develop modules based on their assigned topic. To develop the modules, students first had to conduct a literature review to ensure that they were using the current science in the module content. Each student was required to find 20 peer-reviewed articles on the current science relating to the topic, importance of the topic to families, and tangible best practice tips for families. Teams then compared notes from their literature reviews to create a module script. The script was reviewed by at least one other team and the course teaching assistant (TA), who is also an author on the paper. Following approval and adjustments to the script, teams created their video module. The modules were shown to the entire class, and students voted on the best modules. Modules deemed to be of sufficient quality were further edited by student research assistants (including the course TA) who had more advanced video editing skills and training in family health. The modules were then vetted by the senior author and the larger research lab before posting to the FLE Facebook page.

### Measures

#### Pre- and post-surveys

The measures for each pre- and post-survey were specific to the content of each module topic. Each survey included items relating to participants’ knowledge, attitudes, and self-efficacy to perform skills related to each topic. To develop the items, the research team first examined the literature to determine if there were any existing scales that could be modified for purposes of the current survey. However, given the specificity of each module, this was found to be impractical. Instead, student researchers (the first three authors on this paper) developed the items based on each module. The senior researcher (and corresponding author on this paper) then reviewed each item for coherence, coverage of knowledge/attitudes/self-efficacy, and best practices in wording.

The number of items varied across each module, ranging from six to 11 items. Participants rated each question on a 5-point Likert scale, ranging from 1 (strongly disagree/least confident) to 5 (strongly agree/most confident), depending on the item’s content. Sample items included “I know the difference between listening to understand and listening to respond” (knowledge) and “I feel as though it is important to listen to my teenager without judgment” (attitudes). Self-efficacy was assessed through scenario-based questions in which participants rated their confidence in applying the skills learned from each module. For example, “I have a plan for how I can manage stressors in my life” and “I know how to model healthy eating habits within my family.” In the post-survey, participants rated the quality of each module, including how organized it was, clarity and usefulness of the visuals, ease of understanding, and appropriateness of the module length. Sample items included “This video was well organized” and “The content was easy to understand and follow.” Because each module assessed different content areas and included a different number of knowledge, attitude, and self-efficacy items, the modules were nonparallel and not designed for direct comparison across the different FLE topics. Rather, changes were judged between pre- and post-tests. Apart from the questions on module quality (only included in the post-test), each pre- and post-test survey included the same items on knowledge, attitudes, and self-efficacy to allow for comparison.

#### Family health

Family health was measured using the 10-item Family Health Scale, Short-Form (FHS-SF) (26). Each item was based on a 5-point Likert scale ranging from strongly disagree to strongly agree. Negatively worded items were reverse coded so that higher scores indicated better family health. Items were summed and averaged to create a FHS score at baseline and end-of-study. Baseline measurement of family health was collected from the prior study in 2023. Family health was again measured in the end-of-study survey.

### Data analysis

Data were cleaned and analyzed in Stata 18. Because participants of the same family did not often complete the same module, we did not use dyadic or triadic methods for the analysis. To examine changes in knowledge, attitudes, and self-efficacy between the pre- and post-tests for each module, we used the Stata *signrank* command, which performs the Wilcoxon matched-pairs signed-rank test. This test is the nonparametric alternative to paired t-tests, and it tests the null hypothesis that the median difference between the pre- and post-test was zero. Each item was examined individually as for most modules there were not enough items to create and validate domains. As the number of statistical comparisons could increase the potential for Type I error, we examined effect sizes using the Wilcoxon signed-rank effect size r, including positive, negative, and zero differences. We did not apply a Bonferroni correction for multiple comparisons because the analyses were intended to characterize patterns of change between the pre and post results with distinct, prespecified constructs.

To examine whether module participation was associated with improved family health, we first conducted a Wilcoxon matched pairs signed-rank test comparing family health in 2023 to the end-of-survey responses of family health. We then regressed the end-of-survey family health score on baseline family health and participation in FLE. We conducted two regression modules, one using a dichotomous measure of FLE module participation (1 = completed one or more modules; 0 = participated in no modules) and a second using a continuous variable for module participation (e.g., module participation could range from 0 modules to 10 modules). Finally, we created a variable for change in family health between baseline and end-of-survey. We then conducted two regression modules regressing the change score on the dichotomous and then continuous variable for module participation. To these regression modules we added controls for age in years, sex (1 = female; 0 = male), and race (1 = white; 0 = non-white race).

## Results

### Sample descriptive statistics

Descriptive data of the sample is presented in Table 2. The final sample included 164 participants; most of whom were young adults (*N* = 72) and middle adults (*N* = 75); only 17 older adults participated. The mean age was 39.81 years. Most participants (79.27%) identified as female and 94.57% identified as non-Hispanic White. About one quarter of the sample (27.44%) completed only the baseline survey and did not participate in any video modules. On average, participants watched 3.79 modules. Of those who participated in at least one module (72.56%), they watched an average of 5.26 modules. Nearly 8% of participants watched all 10 modules.

**Table 2:** Descriptive statistics.

Demographic characteristic	*N*	Percent
Age group		
Young Adult	72	43.90
Middle Adults	75	45.73
Older Adults	17	10.37
Female	130	79.27
Age in Years, *M*(*SD*)	164	39.81 (18.15)
White	155	94.57
Participants Who Watched At least One Module	119	72.56
Participants Who Watched All 10 Modules	13	7.92
Number of Modules Watched, *M*(*SD*)	164	3.79 (3.64)

### Engagement by age group (aim 1)

Table 3 includes information on the proportion of the sample who participated in each module for the total sample and by age group. The module topic with the highest participation was reproductive health (48.78%). The topic with the least participation was healing from trauma (28.66%).

**Table 3:** Participation by family life education module.

Module topic	Total sample *N* = 164		Young adults *N* = 72		Middle adults *N* = 75		Older adults *N* = 17	
	*N*	%	*N*	%	*N*	%	*N*	%
Reproductive Health	80	48.78	44	61.11	31	41.33	5	29.41
Positive Adult Experiences	75	45.73	35	48.61	37	49.33	3	17.65
Healthy Habits	59	35.97	31	43.05	26	34.67	2	11.76
Technology Habits in the Family	54	32.93	25	34.72	26	34.67	3	17.65
Mental Health	61	37.19	31	43.05	27	36.00	3	17.65
Healing from Trauma	47	28.66	23	31.94	20	26.67	4	23.53
Parenting Teens	52	31.70	22	30.55	27	36.00	3	17.65
Maintaining Family Relationships across the Lifespan	72	43.90	30	41.67	38	50.67	4	23.53
Communicating about Sensitive Issues	62	36.58	29	40.27	27	36.00	6	35.29
Sibling Rivalry	60	36.58	24	33.33	30	40.00	6	35.30

Percents indicate the number of participants within each group that participated in a module; they are not comparisons across groups.

Among young adults, the module about talking to children about sexual and reproductive health was most popular (61.11%) and the parenting teens module was least popular (30.55%). For adults in midlife, the most popular module was about maintaining family relationships across the lifespan (50.67%) and positive adult experiences (49.33%), while the least popular was healing after trauma (26.67%). While 17 older adults participated in the baseline survey, only seven watched at least one module. The modules with the highest participation from older adults were the modules on how to talk with family about sensitive issues and sibling rivalry (both 35.29% - representing six older adults in each).

### FLE modules and short-term gains in knowledge and skills (aim 2)

Table 4 includes the results of participant increase in knowledge and skills along with their report on the quality of each module. Across all 10 modules participants reported an increase in knowledge from pretest to post-test. Participants reported improvements in all questions relating to knowledge attitudes, and self-efficacy across all modules except for reproductive health (improvements on 4 of 6 items between pre- and post-test; no change on two items) and parenting teens (improvements on 5 of 9 items between pre- and post-test; no change on four items). Absolute effect sizes ranged from 0.00 to 0.68 for reproductive health, 0.61 to 0.82 for positive adult experiences, 0.45 to 0.71 for healthy habits, 0.68 to 0.87 to for technology habits, 0.27 to 0.81 for mental health, 0.59 to 0.88 for healing from trauma, 0.00 to 0.68 for parenting teens, 0.44 to 0.73 for maintaining healthy relationships, 0.47 to 0.84 for communicating about sensitive issues, and 0.53 to 0.86 for sibling rivalry (see Supplementary Table 1 for full results). Participants reported generally high scores on the modules being well-organized (*M* = 4.47–4.91 on a 5-point scale), clear (*M* = 4.44–4.64 on a 5-point scale), and engaging (*M* = 3.88–4.90 on a 5-point scale).

**Table 4:** Participant report on improvement in knowledge, attitudes, and skills and module quality (*N* = *164*).

Module	# of knowledge/ attitude/ self-efficacy items with significant improvement between pre-and post-tests	Module was well-organized *M*(*SD*)	Image quality *M*(*SD*)	Module content was easy to understand *M*(*SD*)	Length of module was satisfactory *M*(*SD*)	Engaging module *M*(*SD*)
Reproductive health *N* = 80	4 of 6	4.49(0.53)	3.91(0.87)	4.46(0.64)	4.18(0.85)	3.89(0.93)
Positive adult experiences *N* = 75	6 of 6	4.46(0.76)	4.33(0.85)	4.59(0.70)	4.28(0.87)	3.95(0.96)
Healthy habits *N* = 59	7 of 7	4.69(0.46)	4.39(0.72)	4.66(0.51)	4.49(0.70)	4.08(0.97)
Technology habits in the family *N* = 54	8 of 8	4.91(0.29)	4.65(0.52)	4.85(0.36)	4.87(0.34)	4.63(0.68)
Mental health *N* = 61	7 of 7	4.57(0.64)	4.21(0.90)	4.59(0.76)	4.39(0.88)	4.15(0.93)
Healing from trauma *N* = 47	7 of 7	4.51(0.80)	4.45(0.69)	4.57(0.80)	4.38(0.92)	4.13(1.06)
Parenting teens *N* = 52	5 of 9	4.54(0.85)	4.21(1.00)	4.50(0.96)	4.54(0.92)	4.23(0.96)
Maintaining family relationships across the lifespan *N* = 72	7 of 7	4.49(0.86)	4.26(9.95)	4.65(0.65)	4.58(0.75)	4.25(0.82)
Communicating about sensitive issues *N* = 62	11 of 11	4.47(0.82)	3.92(1.15)	4.66(0.54)	4.27(0.94)	4.05(0.91)
Sibling rivalry *N* = 60	7 of 7	4.55(0.67)	4.33(0.91)	4.55(0.85)	4.50(0.77)	4.25(0.99)

### Long-term gains in knowledge and family health (aim 3)

Controlling for baseline family health, participating in at least one module was not associated with family health as reported in the post-test, but was trending in the hypothesized direction (*B* = 0.24, *p* = *0*.11, *95% CI*: −0.05 to 0.54) among the 89 participants for whom we had posttest results. When we added controls for age, sex, and race, the results were still non-significant (*B* = 0.26, *p* = *0*.13, *95% CI*: −0.08 to 0.60); giving missing data on some controls, the sample size for this regression model was only 73. There were no differences in family health based on age, sex, or race. We found no differences in family health based on the number of modules completed. In a sensitivity analysis, we also examined whether participating in modules resulted in long-term changes in behaviors associated with the specific modules topics that were measured in the end-of-study survey. No long-term effects were significant.

## Discussion

Examining a pilot FLE program, the study produced findings that are partially supported by the hypotheses across the three study aims. For Aim 1, which examined generational patterns of participation, we had hypothesized that young adults would be most likely to participate (hypothesis 1), but there was fairly equal participation between both young and middle-aged adults, suggesting broader engagement than initially expected in middle-aged adults. However, as expected, few older adults participated. Consistent with hypothesis 2, which assessed short-term knowledge and skill gains following individual module completion; participants reported short-term gains in knowledge and self-efficacy across all 10 modules. Contrary to hypothesis 3, which examined whether module completion would lead to longer-term improvements in family health, these short-term gains did not translate to improved family health scores at the end of the project. However, there were trend-level improvements in family health.

### Engagement and retention in online FLE

Family-based prevention programs often see recruitment rates of 3–35% (27). In our study, 34% of participants that were invited completed at least the baseline survey and/or one module. Thus, engagement in the program was at the upper end for family-based programs. We note that we recruited our sample from a prior sample that had already indicated interest in family issues by engaging in a three-generation study on family health. As such, online FLE recruitment in other samples may be lower. Additionally, participants were given gift cards for each module completed, which further incentivized participation. When funds are available, even small participant incentives (e.g., a $5 Amazon gift card) may be enough to encourage participation in at least one module.

The percent of participants who completed all 10 modules was low (only 8%). Once a participant engaged in at least one module, however, this did lead them to engaging in on average half of the modules. We note that the setting up of this FLE program was such that participants were not asked to complete all modules. Rather, participants were encouraged to complete those modules that they felt were relevant and/or of interest to them. Thus, examining participant dropout for this study would not be appropriate. The modules did not build on each other, but each module was created to stand on its own. Additionally, some modules were not applicable to every life stage. FLE programs that target specific family life stages may see completion of a greater number of modules.

### Generational patterns in participation

In the current study, young adults and middle-aged adults were similarly likely to participate in the FLE modules, but few older adults participated. Between young and middle adults, there were some differences in popularity by module topics. Differences in online module participation by life stage may be due to at least two things: (1) comfort using online tools, and (2) relevance of the topics to the participant’s stage of life.

Both young adults and middle adults appeared to be comfortable navigating the online platform – including use of Facebook and taking surveys and watching videos online. However, older adults were less likely to join the Facebook page in the first place and less likely to participate in each module. This is likely due in part to differences in social media use by age groups. For example, in 2021, Pew Research found that 84% of young adults (18–29 years), 81% of adults 30–49 years, and 73% of adults 50–64% used social media. Only 45% of adults 65 years and older reported using social media (28). However, older age groups also saw the greatest increase in social media use since 2010 (28), indicating that recruiting through social media in older age groups may become less of a barrier over time. In addition, studies on older adult education have shown that engagement increases when programs use easy technology, larger text, and slower pacing (29).

Participation patterns in other studies indicate that engagement increases when the topic of the module correlates to their needs (29). While some major family transitions- such as parenthood, employment changes, and marriage- are widely experienced across adulthood that can sometimes lead to temporary declines in wellbeing, other topics are less relevant to certain life stages (29). Findings from the current study align with these findings. Young adults were perhaps more inclined to watch modules about reproductive health due to wanting to prepare for relationships and family. This aligns with research in emerging adulthood that indicates that young adults are in a developmental period characterized by exploring identity, relationships, and future family roles (30). In contrast, middle adulthood is defined by parenting, managing long-term relationships, and creating a work-life balance. Parenting consists of parent communication skills, message interpretation, and ability to navigate reciprocal parent–child interactions (31). Thus, modules dealing with parenting teens, sensitive issues, or positive adult experiences (PAEs) are especially relevant to this stage of life. On the other hand, older adults have already lived through many of the topics relating to modules such as navigating teen behavior and learning about reproductive health. Of the older adults who did participate in the current study, they were more likely to engage with modules on discussing sensitive issues and sibling rivalry, issues that were relevant to their life course. In the current study, we tried to incorporate a variety of topics, so that at least some topics would be of interest to each age group. Considering these factors, it is understandable that there was a variety of module interest for the different age groups.

### Short-term vs. long-term gains in knowledge, skills, and family health

In the current study, it was found that there were significant short-term gains in knowledge and attitude surrounding family health topics. A study by Méndez-Carbajo & Wolla (32) found that short-form online learning experiences tend to produce measurable short-term gains, even when the content is brief. This pattern mirrors the immediate increases in knowledge and attitudes seen in the current study, suggesting that well organized, self-paced modules can effectively support rapid learning.

However, it is important to note that there were no significant long-term changes reported from the study. One likely explanation is that the participants of the study engaged with each module topic only once, thus removing the opportunity to have repeated exposure. Research on memory and learning indicates that long-term retention typically requires repeated retrieval or repeated exposure, whereas a single learning session produces only short-term gains (33). This suggests that while the modules were effective in producing immediate knowledge gains, their format may not have been sufficient to support sustained behavior or relationship changes over time. In addition to limited exposure, other design factors may have contributed to the lack of long-term effects. For example, the brief length of the modules, while helpful for accessibility, may not have allowed sufficient opportunities to promote lasting change. Thus, the modules may not have provided enough time for long-term behavioral change. Additionally, the use of overall family health as the primary outcome measure may not have been sensitive to more gradual changes. Other long-term indicators, such as communication confidence, parenting self-efficiency, or perceived relationship quality, may have been better suited to capture sustained effects. It is notable that although long-term changes in family health were not significant, results were trending in a positive direction (*p*-values ranging from 0.05 to close to 0.10), which suggests that even with minimal exposure the modules may have had a positive impact on the health of the family. Future research should consider repeated topic exposure that may lead to more long-term outcomes in family life over time and a larger sample size, which may be necessary to have the statistical power to demonstrate longer-term impacts.

### Implications

Taken together, these findings point to several important implications for both the design and delivery of online FLE. First, the short-term gains found in this study suggest that self-paced, brief modules can effectively support learning across family life stages. This style of FLE may be more appropriate for families that are generally functioning well (i.e., “low-risk”) but who want brief exposure to family life topics with evidence-based information. This style of FLE is generally low-cost and college students can be effectively engaged in helping to develop and design content.

Second, to effect longer-term gains it may be more effective to develop a more targeted curriculum that provides repeated exposure on topics specific to the family life stage of participants. For example, it may be more meaningful to develop curriculum that is specific to the needs of a specific family life stage: young adults as they form families of their own, parents of young children or adolescents – or learning to parent young adults, older adults seeking to maintain relationships across generations and navigate life with a different set of responsibilities, and so forth.

Third, families who are more “at-risk” for a variety of reasons may benefit from FLE that provides short and asynchronous modules, as the current FLE curriculum did, but that is also paired with additional supports from certified family life educators or community health workers. Traditional FLE in in-person settings such as schools or community centers, allows the learner to interact with instructors and the content, which produces longer-term results (17). Finding ways to pair asynchronous online content that is available on demand (which may be important for families working non-traditional hours or with childcare concerns) with a support person may elicit the optimal FLE format combining flexible education with opportunities to interact with a trusted expert.

Fourth, all video content in this study was developed by young adult college students (both undergraduate and graduate students), who tend to be highly fluent in digital communication and online media. Most were majoring in public health, though a few were in allied fields such as psychology, family science, and premedical fields. Despite being student-created, the modules were consistently rated as well-organized and easy to understand. This suggests that involving college students in the development of online FLE materials may be valuable both as a service and a learning opportunity for those making and watching the modules. Future programs could intentionally use student creators to have current technology and provide experiential learning opportunities.

### Limitations

Some limitations of this study are important to note. First, because participants self-selected into the study and because they were recruited from a larger study that was initiated in the U. S. Intermountain West and had more limited demographic diversity, they may not be generalizable to the U. S. population at large. Future studies could try to recruit participants from a wider range of geographical and cultural backgrounds. Relatedly, participants also self-selected into the Facebook group and which and how many modules to watch. Consequently, even within the larger sample there was participant bias of those who were willing to use social media and who had interest in the topics. This may be a similar bias of any FLE that is offered over social media and allows participants to self-select which modules they will watch. However, the results may not be generalizable to participants who are, for example, court ordered to participate in FLE or where participant incentives are not offered.

Second, only a few older adults opted into the study making it difficult to infer any of the study findings to older adult populations. The results do indicate that more novel recruitment strategies may be needed to recruit older adults.

Third, while the initial short-term outcomes of the study were consistently measured (pre- and post-tests taken immediately before and after the completion of the modules), there was less consistency when participants watched each module. The modules were available on demand, once posted, for the full length of the study. While many participants watched the modules shortly after they were posted, some watched several modules in a shorter period closer to the end of the study. Thus, the examination for the period for intermediate/long-term changes (measured through family health) would have varied across participants.

Fourth, due to the specificity of each module, we were unable to use validated survey measures for the pre and post-tests. We carefully constructed each survey, but we did not have time to do extended pilot or field testing of the items. Relatedly, participant changes in knowledge, attitudes, self-efficacy, and family health were based on self-report. Studies that observe changes in parenting and family life behavior would be beneficial.

Fifth, as this was a pilot study, we did not include any comparison or control groups. It would be valuable to conduct a study comparing groups using the asynchronous modules with groups attending synchronous online modules and groups attending more traditional in-person FLE. While we were able to establish pre/post-test positive gains in knowledge, attitudes, and self-efficacy, causality cannot be inferred with a non-experimental design.

Sixth, participants were recruited from a three-generation study. In the current study, generally only one member from the same family triad participated in a given module (61% of families represented only had one member participate and of the 39% of families with two or three members represented, the amount of module overlap was minimal). As such we did not feel it was practical to conduct multilevel analyses to account for statistical non-independence given the small number of families having multiple members participate in the same module. However, as there were a few instances when two members of the same family triad participated in the same module, although we believe the impact to be minimal on the overall findings, we cannot assume complete statistical independence.

Finally, the senior author on the study was trained as a public health educator and does work in family health. Many of the principles of health education overlap with family life education best practices. However, none of the authors were certified family life educators.

## Conclusion

The findings of this pilot FLE program highlight that short, online FLE modules accessible to families on demand can lead to short-term gains in knowledge, attitudes, and self-efficacy for young and middle-aged adults. Longer-term gains in family health were not found in this study indicating that short modules without follow-up content may not lead to long-term gains in family health. Thus, additional modules may be helpful in refreshing information for families and helping them to convert short-term gains into long-term habits.

An additional finding of this study was the inclusion of young adult college students in creating the FLE content. Involving young adults as content creators for online FLE can help with incorporating the latest technologies into FLE and provides students with hands-on opportunities to apply knowledge and skills they are learning in the classroom to real world situations.

Finally, families face a variety of challenges throughout the life course. Accessible FLE is important to help families gain information and learn strategies for maintaining healthy habits and developing resilience through the ups and downs of family life. The current study tested whether online video modules could help increase knowledge, attitudes, and self-efficacy surrounding several family life topics. The study also tested whether online modules would be able to increase learning throughout different life stages, from young adults to older adult family members. The modules created were designed to be conducive for self-paced education to different lifestyles. Overall, pilot findings suggest that online, self-paced modules are effective in producing immediate knowledge gains among adults in different stages of family life.

## Data Availability

The raw data are available from the corresponding author upon reasonable request and approval of the appropriate human ethics committees.
