# Supplementary material for: Evaluating online family life education modules across family stages: a pilot study
Source: Front Public Health. 2026 Sep 17;14:1909217. doi: 10.3389/fpubh.2026.1909217 (PMC13627363; doi:10.3389/fpubh.2026.1909217)
Supplement: Supplementary file 1 [file Table_1.docx]

| **Supplemental Table 1. Wilcoxon signed-rank effect sizes (r) for family life education changes (pre and post modules) in knowledge, attitudes, and self-efficacy** | | | | | | | | | | |
| --- | --- | --- | --- | --- | --- | --- | --- | --- | --- | --- |
| **Module** | **Variable *** | **Pre**  **Mean** | **Post**  **Mean** | **N (all pairs)** | **Pre > Post** | **Pre < Post** | **No Change (Zero)** | **z** | **p** | **Absolute Effect Size (r)** |
| **Reproductive Health** | plan | 3.94 | 4.25 | 80 | 2 | 18 | 60 | -3.61 | 0.000 | 0.40 |
| **Reproductive Health** | comfort | 4.18 | 4.41 | 80 | 3 | 15 | 62 | -2.86 | 0.004 | 0.32 |
| **Reproductive Health** | age | 3.76 | 4.50 | 80 | 2 | 43 | 35 | -6.08 | 0.000 | 0.68 |
| **Reproductive Health** | import | 4.85 | 4.89 | 80 | 4 | 4 | 72 | -0.02 | 0.985 | 0.00 |
| **Reproductive Health** | likely | 3.79 | 3.78 | 80 | 5 | 7 | 68 | -0.52 | 0.601 | 0.06 |
| **Reproductive Health** | resource | 3.48 | 3.69 | 80 | 3 | 18 | 59 | -3.29 | 0.001 | 0.37 |
| **Positive Adult Experiences** | aces | 3.51 | 4.84 | 75 | 1 | 52 | 22 | -6.80 | 0.000 | 0.79 |
| **Positive Adult Experiences** | paes | 3.21 | 4.80 | 76 | 2 | 63 | 11 | -7.02 | 0.000 | 0.80 |
| **Positive Adult Experiences** | counter | 2.96 | 4.66 | 76 | 1 | 65 | 10 | -7.18 | 0.000 | 0.82 |
| **Positive Adult Experiences** | resil | 3.88 | 4.62 | 76 | 3 | 45 | 28 | -6.04 | 0.000 | 0.69 |
| **Positive Adult Experiences** | create | 3.75 | 4.53 | 73 | 2 | 41 | 30 | -5.92 | 0.000 | 0.69 |
| **Positive Adult Experiences** | manage | 3.95 | 4.48 | 73 | 1 | 30 | 42 | -5.20 | 0.000 | 0.61 |
| **Healthy Habits** | eat | 4.61 | 4.93 | 59 | 2 | 17 | 40 | -3.45 | 0.001 | 0.45 |
| **Healthy Habits** | modeat | 4.19 | 4.75 | 59 | 1 | 27 | 31 | -4.90 | 0.000 | 0.64 |
| **Healthy Habits** | physic | 4.14 | 4.73 | 59 | 1 | 28 | 30 | -5.00 | 0.000 | 0.65 |
| **Healthy Habits** | mental | 3.69 | 4.46 | 59 | 1 | 35 | 23 | -5.46 | 0.000 | 0.71 |
| **Healthy Habits** | encourag | 3.71 | 4.46 | 59 | 3 | 35 | 21 | -4.91 | 0.000 | 0.64 |
| **Healthy Habits** | physiclife | 3.64 | 4.49 | 59 | 2 | 35 | 22 | -5.40 | 0.000 | 0.70 |
| **Healthy Habits** | wellbeing | 3.78 | 4.61 | 59 | 1 | 33 | 25 | -5.32 | 0.000 | 0.69 |
| **Technology Habits in the Family** | screenhab | 3.60 | 4.51 | 53 | 0 | 34 | 19 | -5.69 | 0.000 | 0.78 |
| **Technology Habits in the Family** | mediause | 2.74 | 4.75 | 53 | 0 | 47 | 6 | -6.30 | 0.000 | 0.87 |
| **Technology Habits in the Family** | encourag | 3.57 | 4.66 | 53 | 0 | 42 | 11 | -6.22 | 0.000 | 0.85 |
| **Technology Habits in the Family** | parental | 4.08 | 4.74 | 53 | 0 | 27 | 26 | -5.15 | 0.000 | 0.71 |
| **Technology Habits in the Family** | communic | 3.58 | 4.40 | 53 | 2 | 30 | 21 | -4.96 | 0.000 | 0.68 |
| **Technology Habits in the Family** | plan | 3.45 | 4.28 | 53 | 1 | 30 | 22 | -5.16 | 0.000 | 0.71 |
| **Technology Habits in the Family** | screentime | 3.58 | 4.38 | 53 | 0 | 30 | 23 | -5.39 | 0.000 | 0.74 |
| **Technology Habits in the Family** | plantalk | 3.43 | 4.38 | 53 | 1 | 35 | 17 | -5.56 | 0.000 | 0.76 |
| **Mental Health** | signs | 3.95 | 4.48 | 61 | 2 | 25 | 34 | -4.45 | 0.000 | 0.57 |
| **Mental Health** | sources | 3.75 | 4.44 | 61 | 4 | 30 | 27 | -4.48 | 0.000 | 0.57 |
| **Mental Health** | tech | 3.02 | 4.33 | 61 | 0 | 44 | 17 | -6.36 | 0.000 | 0.81 |
| **Mental Health** | support | 3.66 | 4.57 | 61 | 0 | 37 | 24 | -5.96 | 0.000 | 0.76 |
| **Mental Health** | famplan | 3.31 | 4.18 | 61 | 3 | 35 | 23 | -5.12 | 0.000 | 0.66 |
| **Mental Health** | talk | 3.87 | 4.25 | 61 | 14 | 29 | 18 | -2.13 | 0.034 | 0.27 |
| **Mental Health** | plansource | 3.11 | 4.13 | 61 | 1 | 39 | 21 | -5.90 | 0.000 | 0.76 |
| **Healing from Trauma** | know | 4.39 | 4.80 | 46 | 0 | 16 | 30 | -3.99 | 0.000 | 0.59 |
| **Healing from Trauma** | impact | 3.89 | 4.67 | 46 | 2 | 26 | 18 | -4.54 | 0.000 | 0.67 |
| **Healing from Trauma** | build | 3.48 | 4.48 | 46 | 0 | 33 | 13 | -5.59 | 0.000 | 0.82 |
| **Healing from Trauma** | heal | 2.72 | 4.50 | 46 | 0 | 43 | 3 | -5.94 | 0.000 | 0.88 |
| **Healing from Trauma** | plan | 3.04 | 4.13 | 45 | 1 | 31 | 13 | -5.04 | 0.000 | 0.75 |
| **Healing from Trauma** | methods | 2.89 | 4.22 | 45 | 1 | 34 | 10 | -5.41 | 0.000 | 0.81 |
| **Healing from Trauma** | trust | 3.73 | 4.49 | 45 | 1 | 26 | 18 | -4.77 | 0.000 | 0.71 |
| **Parenting Teens** | warmth | 4.79 | 4.85 | 52 | 1 | 4 | 47 | -1.34 | 0.180 | 0.19 |
| **Parenting Teens** | present | 4.79 | 4.83 | 52 | 2 | 6 | 44 | -1.37 | 0.171 | 0.19 |
| **Parenting Teens** | listen | 4.83 | 4.77 | 52 | 4 | 4 | 44 | 0.03 | 0.977 | 0.00 |
| **Parenting Teens** | develop | 4.75 | 4.83 | 52 | 2 | 8 | 42 | -1.85 | 0.064 | 0.26 |
| **Parenting Teens** | rules | 3.85 | 4.29 | 52 | 5 | 21 | 26 | -3.24 | 0.001 | 0.45 |
| **Parenting Teens** | indepen | 4.10 | 4.63 | 52 | 2 | 24 | 26 | -4.33 | 0.000 | 0.60 |
| **Parenting Teens** | success | 3.71 | 4.17 | 52 | 4 | 20 | 28 | -3.27 | 0.001 | 0.45 |
| **Parenting Teens** | bound | 3.46 | 4.27 | 52 | 1 | 27 | 24 | -4.89 | 0.000 | 0.68 |
| **Parenting Teens** | encour | 3.90 | 4.48 | 52 | 2 | 25 | 25 | -4.45 | 0.000 | 0.62 |
| **Maintaining Healthy Relationships** | genera | 4.23 | 4.69 | 71 | 3 | 31 | 37 | -4.71 | 0.000 | 0.56 |
| **Maintaining Healthy Relationships** | connect | 4.30 | 4.66 | 71 | 4 | 24 | 43 | -3.74 | 0.000 | 0.44 |
| **Maintaining Healthy Relationships** | benefit | 4.20 | 4.69 | 71 | 3 | 28 | 40 | -4.43 | 0.000 | 0.53 |
| **Maintaining Healthy Relationships** | risks | 3.37 | 4.37 | 71 | 6 | 44 | 21 | -5.42 | 0.000 | 0.64 |
| **Maintaining Healthy Relationships** | distance | 3.73 | 4.59 | 71 | 2 | 44 | 25 | -6.13 | 0.000 | 0.73 |
| **Maintaining Healthy Relationships** | plan | 3.21 | 4.15 | 71 | 5 | 45 | 21 | -5.56 | 0.000 | 0.66 |
| **Maintaining Healthy Relationships** | caregiver | 3.79 | 4.30 | 71 | 6 | 31 | 34 | -4.04 | 0.000 | 0.48 |
| **Communicating about Sensitive Issues** | listen | 4.15 | 4.81 | 54 | 0 | 30 | 24 | -5.43 | 0.000 | 0.74 |
| **Communicating about Sensitive Issues** | core | 3.26 | 4.61 | 54 | 0 | 42 | 12 | -6.15 | 0.000 | 0.84 |
| **Communicating about Sensitive Issues** | tension | 4.07 | 4.61 | 54 | 5 | 25 | 24 | -3.75 | 0.000 | 0.51 |
| **Communicating about Sensitive Issues** | differ | 4.09 | 4.80 | 54 | 1 | 29 | 24 | -5.08 | 0.000 | 0.69 |
| **Communicating about Sensitive Issues** | aware | 3.89 | 4.74 | 54 | 1 | 36 | 17 | -5.51 | 0.000 | 0.75 |
| **Communicating about Sensitive Issues** | empathy | 4.43 | 4.83 | 54 | 3 | 19 | 32 | -3.44 | 0.001 | 0.47 |
| **Communicating about Sensitive Issues** | confident | 3.80 | 4.43 | 54 | 4 | 27 | 23 | -4.20 | 0.000 | 0.57 |
| **Communicating about Sensitive Issues** | perspect | 3.89 | 4.56 | 54 | 4 | 28 | 22 | -4.21 | 0.000 | 0.57 |
| **Communicating about Sensitive Issues** | try | 3.39 | 4.78 | 54 | 1 | 46 | 7 | -6.20 | 0.000 | 0.84 |
| **Communicating about Sensitive Issues** | intention | 3.91 | 4.65 | 54 | 3 | 31 | 20 | -4.83 | 0.000 | 0.66 |
| **Communicating about Sensitive Issues** | strategy | 3.30 | 4.54 | 54 | 0 | 40 | 14 | -6.06 | 0.000 | 0.82 |
| **Sibling Rivalry** | rivalry | 4.22 | 4.88 | 58 | 2 | 30 | 26 | -4.95 | 0.000 | 0.65 |
| **Sibling Rivalry** | know | 3.74 | 4.52 | 58 | 1 | 34 | 23 | -5.52 | 0.000 | 0.72 |
| **Sibling Rivalry** | benefits | 2.74 | 4.22 | 58 | 1 | 45 | 12 | -6.26 | 0.000 | 0.82 |
| **Sibling Rivalry** | gener | 3.86 | 4.67 | 58 | 1 | 32 | 25 | -5.35 | 0.000 | 0.70 |
| **Sibling Rivalry** | plan | 3.16 | 4.28 | 58 | 2 | 46 | 10 | -6.15 | 0.000 | 0.81 |
| **Sibling Rivalry** | handle | 2.83 | 4.28 | 58 | 0 | 49 | 9 | -6.54 | 0.000 | 0.86 |
| **Sibling Rivalry** | mend | 3.86 | 4.33 | 58 | 3 | 24 | 31 | -4.07 | 0.000 | 0.53 |
|  |  |  |  |  |  |  |  |  |  |  |
| **Notes** | Counts reflect signrank pre = post: Pre > Post is Stata Positive; Pre < Post is Stata Negative. | | | | | | | | | |
|  | Table prepared with the help of ChatGPT. | | | | | | | | | |
|  | *Full survey items are available from the corresponding author upon request. | | | | | | | | | |
